# The risk of neoplasms in appendiceal abscess: what emergency surgeons should know

**DOI:** 10.3389/fonc.2026.1767622

**Published:** 2026-05-04

**Authors:** Giulia Montori, Mauro Podda, Alessio Giordano, Giuseppa Procida, Vittoria Butera, Valentina Murzi, Paola Fugazzola, Carlo Bergamini, Fausto Catena, Luca Ansaloni, Adolfo Pisanu, Ferdinando Agresta

**Affiliations:** 1 Department of General Surgery, Ulss2 Marca Trevigiana, Vittorio Veneto, Treviso, Italy; 2 Department of Surgical Science, University of Cagliari, Cagliari, Italy; 3 Emergency Surgery Unit, Careggi University Hospital, Florence, Italy; 4 Department of Clinical, Surgical, Diagnostic & Pediatric Sciences, University of Pavia, Pavia, Italy; 5 Department of Medical and Surgical Sciences, Alma Mater Studiorum University of Bologna, Bologna, Italy

**Keywords:** appendiceal abscess, appendiceal tumors, complicated acute appendicitis, follow-up, interval appendectomy, management strategies

## Abstract

**Introduction:**

A subset of patients with acute appendicitis presents with complicated disease characterized by periappendiceal abscess. Although appendiceal neoplasms are rare in uncomplicated appendicitis, recent evidence suggests a higher incidence in patients with appendicitis complicated by abscess, particularly in older adults. Because preoperative imaging has limited accuracy for tumor detection, the optimal management strategy remains debated. This narrative review summarizes current evidence on the incidence, histology, diagnostic limitations, and management implications of appendiceal neoplasms in patients with appendiceal abscess.

**Methods:**

A literature search was conducted in MEDLINE (PubMed) and the Cochrane Central Register of Controlled Trials from inception to November 2025. Eligible studies included randomized controlled trials, prospective and retrospective cohort studies, and case–control studies addressing complicated acute appendicitis with periappendiceal abscess and the incidence or characteristics of appendiceal neoplasms. Data were synthesized qualitatively through a narrative approach.

**Results:**

Primary studies show a low incidence of appendiceal neoplasms in uncomplicated acute appendicitis, generally below 2%. Patients with appendicitis complicated by periappendiceal abscess demonstrate substantially higher neoplasm rates, reaching 10–15% in population-based cohorts and selected interval appendectomy series. Most tumors are diagnosed incidentally on postoperative histopathology. Neuroendocrine tumors are more frequently identified in patients with uncomplicated acute appendicitis, whereas low-grade appendiceal mucinous neoplasms predominate in cases complicated by periappendiceal abscess. Invasive adenocarcinoma is also reported across both clinical presentations, with a higher prevalence in older patients. Age is the strongest predictor of underlying neoplasia, with a marked risk increase above 35–40 years. Pediatric studies consistently show a negligible risk of malignancy, with rare incidental neuroendocrine tumors and no association between appendiceal abscess and epithelial cancer.

**Conclusions:**

Appendiceal abscess in adults aged 35 years or older is associated with a clinically relevant risk of underlying neoplasia that cannot be reliably excluded preoperatively. Interval appendectomy after successful non-operative management provides diagnostic certainty and oncologic safety in this population. Standardized imaging-based definitions and international registries focused on CT-confirmed appendiceal abscess are needed to refine incidence estimates and optimize management strategies.

## 1 Introduction

Acute appendicitis is one of the most common causes of acute abdomen in adults, with an incidence of about 90–100 cases/100,000 inhabitants/year in developed countries (1). Both mean age at diagnosis and incidence rate seem to increase over time. A dissimilar epidemiologic trend has been reported between complicated and uncomplicated appendicitis suggesting a different pathophysiology of these diseases (2).

According to the World Society of Emergency Surgery (WSES) guidelines, uncomplicated acute appendicitis is defined as the inflammation of the appendix with hyperemia and/or phlegmon, whereas complicated acute appendicitis is the inflammation of the appendix with extended gangrene/necrosis, perforation, abscess or diffuse peritonitis (3).

Clinically, the result of a walled-off appendiceal perforation is an appendiceal mass represented by an inflammatory tumor, consisting of the inflamed appendix, its adjacent viscera and the greater omentum forming a phlegmon or an abscess. The occurrence of a periappendiceal abscess may or not coexist with a perforated gangrenous appendicitis, and a clinically palpable mass may represent an advanced stage of the inflammatory process (4).

The prevalence of complicated appendicitis presenting with a periappendiceal abscess ranges between 3% and 10% (5). Similar to acute diverticulitis showing an increased neoplasm risk associated with diverticular abscess, the reported appendiceal tumor risk associated with complicated acute appendicitis has been reported to be markedly higher in the forms with periappendiceal abscess compared with uncomplicated acute appendicitis (6).

In general, the reported overall risk of an underlying malignant neoplasm in acute appendicitis is very low, varying between 0.7 and 3% (7). However, there are many recent studies reporting an alarming rate of appendiceal neoplasms detected at interval appendectomy in patients with previous periappendiceal abscess.

A recent randomized controlled trial comparing interval appendectomy and follow-up with magnetic resonance imaging in patients with initial successful antibiotic treatment of periappendiceal abscess was prematurely terminated based on high tumor rate in the interval appendectomy group, with an overall appendiceal neoplasm incidence of 20%, and all of these patients were over 40 years of age (8). Furthermore, the incidence seems to increase in case of larger appendiceal diameter, and in patients with severe obesity (4, 9–11). In most of patients, moreover, the diagnosis is incidental, with the tumor found only at definitive histological evaluation, done after an emergency appendectomy for acute appendicitis.

The aim of this narrative review is to summarize the findings of the literature on this subject, particularly in light of the risk of appendiceal neoplasm in patients with acute appendicitis complicated by abscess, and to suggest therapeutic strategies for these patients.

## 2 Materials and methods

The literature search was conducted in accordance with the Preferred Reporting Items for Systematic Reviews and Meta-Analyses (PRISMA) 2020 guidelines (12). Although this study represents a narrative review, PRISMA principles were applied to structure the search strategy and selection process. A systematic literature search was performed in MEDLINE (via PubMed) and the Cochrane Central Register of Controlled Trials (CENTRAL) from database inception to November 2025. The complete search strategy is reported in **Table 1**. Additional manual searches were conducted by reviewing reference lists of relevant articles and previously published systematic reviews to identify further eligible studies.

**Table 1:** Strategy used for the primary literature search.

(“complicated acute appendicitis” OR “acute appendicitis” OR appendicitis OR “appendicular abscess” OR “appendiceal abscess”) AND (“laparoscopic appendectomy” OR “laparoscopic appendicectomy” OR appendectomy OR appendicectomy) AND (“non-operative treatment” OR “non-operative management” OR NOM OR “conservative treatment”). (“uncomplicated acute appendicitis” OR “uncomplicated appendicitis” OR “acute appendicitis” OR appendicitis) AND (“interval appendectomy” OR “interval appendicectomy”). (“complicated acute appendicitis” OR “acute appendicitis” OR appendicitis OR “appendicular abscess” OR “appendiceal abscess”) AND (“interval appendectomy” OR “interval appendicectomy”). (“uncomplicated acute appendicitis” OR “acute appendicitis” OR appendicitis) AND (“non-operative treatment” OR “non-operative management” OR NOM OR “conservative treatment”) AND (follow-up).

### 2.1 Inclusion criteria

Studies were considered eligible if they met the following criteria:

- Literature addressing the management of complicated acute appendicitis with periappendiceal abscess and the incidence, diagnosis, or risk factors of appendiceal neoplasms in the context of periappendiceal abscess.
- Original research articles, including randomized controlled trials, non-randomized controlled trials, prospective or retrospective cohort studies, and case–control studies. Review articles were not included as primary evidence but were examined to support background context and to retrieve additional sources.
- Articles published in English.

### 2.2 Exclusion criteria

Publications without accessible full text, case reports, conference abstracts, letters, expert opinions, and documents lacking primary data were excluded.

### 2.3 Study selection and data management

Two reviewers [MP, GM] independently screened titles and abstracts, followed by full-text evaluation of potentially eligible studies. Discrepancies were resolved through discussion. Extracted data included study design, population, sample size, appendicitis type, incidence and histologic subtypes of appendiceal neoplasms.

### 2.4 Synthesis

A qualitative, narrative synthesis was performed. The analysis focused on the following predefined thematic areas:

- Incidence of appendiceal neoplasms in uncomplicated acute appendicitis;
- Incidence of appendiceal neoplasms in acute appendicitis complicated by abscess or phlegmon;
- Preoperative diagnosis of appendiceal neoplasms in acute appendicitis;
- Histologic subtypes of appendiceal neoplasms;
- Effect of patient age on the risk of underlying neoplasm.
- Evidence in children

Data extracted from the eligible studies were summarized descriptively within each thematic domain.

## 3 Results

### 3.1 Review of the existing secondary literature

In the systematic review by Teixeira et al., which included 13,244 patients with acute appendicitis, appendiceal tumors were identified in approximately 0.9–1.4% of appendectomy specimens in patients with uncomplicated appendicitis, while the rate of neoplasm varies from 10 to 29% in patients presenting appendiceal inflammatory mass (13). Solis-Pazmiño et al. analyzed 4,962 patients from four comparative studies and reported an overall appendiceal neoplasm incidence of 1.9% across complicated and uncomplicated appendicitis (14). Peltrini et al., focusing specifically on patients undergoing interval appendectomy after complicated appendicitis with appendiceal mass (abscess and/or phlegmon), found a substantially higher pooled prevalence of neoplasms (11%) in this selected subgroup, indicating that epidemiological risk is not uniform across all clinical presentations (15).

Appendiceal tumors encompass neuroendocrine tumors (NET), mucinous neoplasms, nonmucinous adenocarcinomas, mixed adeno-neuroendocrine tumors (MANEC), and other epithelial lesions. Teixeira et al. describe NET and low-grade appendiceal mucinous neoplasms (LAMN) as the most frequent histologic subtypes among tumors discovered in patients with an appendiceal inflammatory mass, with carcinoma and pseudomyxoma-related lesions representing a smaller fraction (13). The meta-analysis by Solis-Pazmiño et al. provides quantitative histologic distribution: NET accounted for 49.2%, nonmucinous adenocarcinoma for 24.2%, MANEC for 11.4%, and mucinous adenocarcinoma for 4.4% of tumors across complicated and uncomplicated appendicitis (14). Peltrini et al. found a different profile: LAMN in 43%, adenocarcinoma in 29%, NET in 21%, globet cell carcinoma in 13%, and adenoma/serrated lesions in 20% of cases (15). In the pediatric population, the histologic spectrum is narrower. Hall et al. reported that virtually all tumors found after interval appendectomy in children with appendix mass were carcinoid/NET, with an estimated incidence of carcinoid of 0.9% at appendectomy (16).

Hajibandeh et al. showed that adults with acute appendicitis have an age-dependent increase in the risk of right-sided colon cancer. In patients over 40 years, the pooled incidence was 1.04%, corresponding to a 10-fold higher risk compared with the general population. Among patients over 50 years, the pooled incidence increased to 1.85%, with a standardized risk ratio of 11.82. The review did not provide stratified analyses according to appendicitis severity (17). Similarly, Forsyth et al. noted that adults over 40 years with an appendix mass have an elevated risk of underlying colorectal cancer, reported between 5.9% and 8% (18).

In the review by Peltrini et al., patients diagnosed with appendiceal neoplasms at interval appendectomy showed a consistent age pattern, with most studies reporting mean ages between the mid-50s and early 60s. Across the included series, the mean age at neoplasm diagnosis ranged from 49 to 62 years, and two studies demonstrated significantly higher neoplasm rates in patients over 40 or over 70 years (15). Solis-Pazmiño et al. reported an overall mean age of 43.6 years among 3,701 patients included in their analysis (range 16–94 years). One study within the review showed that patients with complicated appendicitis and appendiceal neoplasms were significantly older than those with uncomplicated appendicitis and neoplasms (52.9 vs 40.4 years) (14).

### 3.2 Evidence from original research

Three hundred forty-seven records were identified through database searching. After removal of two duplicates, 345 records underwent title and abstract screening. Of these, 291 records were excluded as not relevant to the scope of the review.

**Figure 1:**
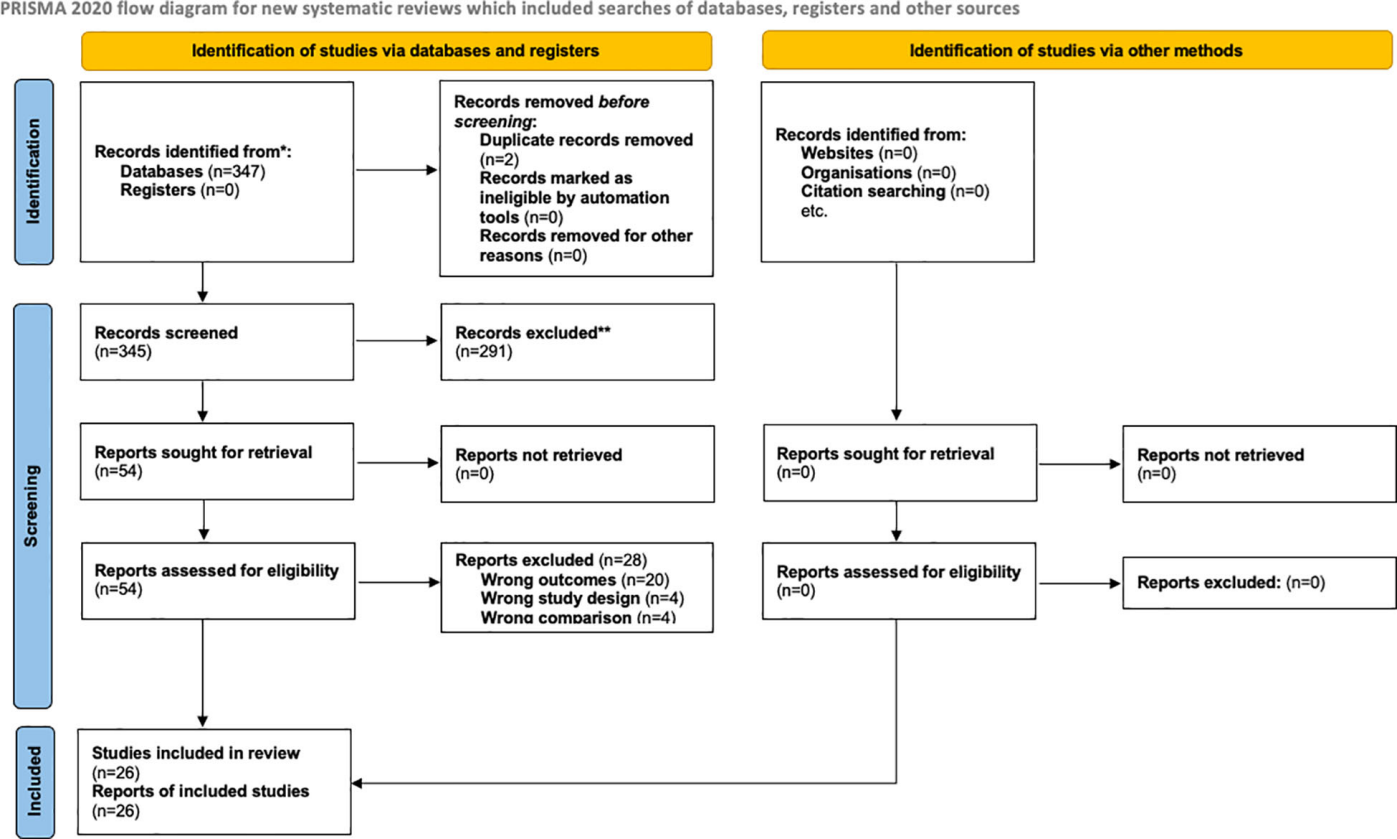
PRISMA 2020 flow diagram for new systematic reviews which included searches of databases, registers and other sources.

A total of 54 full-text articles were assessed for eligibility, and 28 were subsequently excluded for the following reasons: inappropriate outcomes (*n=20*), unsuitable study design (*n=4*), or lack of a relevant comparison (*n=4*). Ultimately, 26 studies met the inclusion criteria and were incorporated into the narrative synthesis (5, 8, 19–42) (**Table 2**; **Figure 1**).

**Table 2:** Summary of the primary studies included in the review.

First author	Study design	Population	Sample size	Appendicitis type	Tumor incidence	Histologic subtype
Salminen 2025 (5)	Prospective cohort study	Adults	370	Periappendiceal abscess (imaging-confirmed)	14.3% (53/370)	21 LAMN, 20 adenocarcinoma, 8 adenoma, and 5 NET. Tumor risk increased at an age cut-off of 35 years
Flum 2023	Randomized clinical trial	Adults	2062	Uncomplicated appendicitis (imaging-confirmed). Appendicoliths included	0.6% (12/2062)	5 patients with NET, 2 patients with SSA, 3 patients with LAMN, and 2 patients with adenocarcinoma
Hellmann 2024	Retrospective cohort study	Children	2826	Complicated appendicitis	0.02% (1/2826)	1 unspecified tumor
Rashid 2024 (21)	Retrospective cohort study	Adults	211	Complicated appendicitis (imaging-confirmed)	3.8% (8/211)	2 appendiceal adenocarcinoma 1 LAMN, 4 NET, 1 report not available. The mean age of patients diagnosed with appendiceal neoplasm was 67.5 years
Ramadan 2023 (22)	Retrospective cohort study	Adults	239	Complicated appendicitis with phlegmon or abscess (imaging-confirmed)	5% (12/239)	4 adenocarcinoma (1 pseudomyxoma), 7 LAMN, 1 NET. Most patients (75%) with appendiceal malignancy were >40 years of age
Sugimoto 2022 (23)	Retrospective cohort study	Adults	297	Complicated appendicitis with phlegmon or abscess (imaging-confirmed)	4.7% (14/297)	6 adenocarcinoma, 3 LAMN, 1 adenoma, 2 NET, 2 globet cell tumor. among patients aged ≥ 60 years, the incidence of appendiceal tumor was significantly higher
Hayes 2021 (24)	Retrospective cohort study	Adults	402	Complicated appendicitis with perforation, abscess, and/or phlegmon (imaging-confirmed)	9% (36/402)	17 LAMN, 7 NET, 7 adenoma, 4 adenocarcinoma, 1 adenocarcinoma ex goblet cell carcinoid. Patients with tumor were older (>56 years)
Farr 2020	Retrospective cohort study	Children	500	Perforated appendicitis	1.2% (6/500)	6 carcinoid tumor
Naar 2020 (26)	Retrospective cohort study	Adults	3293	Perforated and gangrenous appendicitis	1.5% (48/3293)	29 adenocarcinoma, 18 NET, 1 B-cell lymphoma. Patients >40 years old had the highest tumor incidence
Son 2020 (27)	Retrospective cohort study	Adults	2013	Complicated appendicitis with phlegmon or abscess (imaging-confirmed)	1.8% (36/2013)	27 LAMN, 3 mucinous adenocarcinoma, 2 tubular adenoma, 2 NET, 1 hyperplastic polyp (1.8%), 1 SSA. Patients >60 years old had the highest tumor incidence
DeJonge 2019 (28)	Retrospective cohort study	Adults	64	Complicated appendicitis with abscess (imaging-confirmed)	11% (7/64 patients) 17.2% (11/64 tumors)	3 adenocarcinoma, 2 LAMN, 2 NET, 2 globet cell carcinoid, 1 SSA, 1 signet-ring cell carcinoma
Fouad 2019	Retrospective cohort study	Children	149	Complicated appendicitis with phlegmon or abscess (imaging-confirmed)	–	–
Lietzen 2019	Retrospective cohort study	Adults	472 patients with appendiceal tumors	Complicated appendicitis with abscess (imaging-confirmed)	102 patients had complicated appendicitis (41%). 3.2% of patients with complicated appendicitis had tumor. Tumor risk in patients with appendiceal abscess was 4.9%.	232 NET, 52 globet cell, 65 mucinous tumor or pseudomyxoma peritonei, 123 adenocarcinoma. Patients >46 years old had the highest tumor incidence
Mallinen 2019	Randomized clinical trial	Adults	60	Complicated appendicitis with abscess (imaging-confirmed)	20% (12/60)	5 LAMN, 2 adenocarcinoma, 3 SSA, 1 mucinous cystadenoma, 1 globet cell carcinoid. Patients >40 years old had the highest tumor incidence (mean age at diagnosis 56 years)
Mima 2019	Retrospective cohort study	Adults (Elderly)	50	Complicated appendicitis with abscess (imaging-confirmed)	8.0% (4/50)	2 adenocarcinoma, 1 NET, 1 mucinous cystadenoma. Included patients were >70 years old
Siddharthan 2019 (32)	Retrospective cohort study	Adults	1937 patients with appendiceal cancer	Appendiceal cancer confirmed at pathology	–	Mean age at diagnosis was 75 years
Hall 2017 (33)	Randomized clinical trial	Children	106	Complicated appendicitis with appendix mass (imaging-confirmed)	–	–
Wright 2015 (34)	Retrospective cohort study	Adults	89	Complicated appendicitis with perforation	12.0% (11/89)	6 LAMN, 4 carcinoid tumor, 1 adenocarcinoma. The rate of neoplasm in patients over age 40 was 16%
Fawkner Corbett 2014 (35)	Retrospective cohort study	Children	69	Complicated appendicitis with appendiceal mass (imaging-confirmed)	–	–
Svensson 2014 (36)	Retrospective cohort study	Children	89	Complicated appendicitis with appendiceal abscess (imaging-confirmed)	–	–
Furman 2013 (37)	Retrospective cohort study	Adults	376	Presumed acute appendicitis	3.7% (14/376)	6 mucinous adenocarcinoma, 3 mucinous cystadenoma, 2 carcinoid tumor, 2 metastatic endocervical and lung lesions, 1 lymphoma. The mean age of all patients with appendiceal tumors was 49 years (range, 35–74 years)
Carpenter 2012 (38)	Retrospective cohort study	Adults	18	Complicated appendicitis with appendiceal abscess (imaging-confirmed)	28% (5/18)	1 mucinous adenocarcinoma, 1 carcinoid, 1 tubulovillous adenoma with high-grade dysplasia, 1 adenocarcinoma (colonic type). Mean age of patients with malignancy was 60 years
You 2012 (39)	Retrospective cohort study	Adults	15	Complicated appendicitis with appendiceal abscess (imaging-confirmed)	–	–
Lai 2006 (40)	Retrospective cohort study	Adults	165	Complicated appendicitis with appendiceal mass (phlegmon/abscess) (imaging-confirmed)	4.8% (8/165)	5 colon cancer, 3 mucinous tumor/mucocele
Eriksson 2003	Retrospective cohort study	Adults	38	Complicated appendicitis with appendiceal abscess or chronic appendicitis (imaging-confirmed)	2.6% (1/38)	1 adenocarcinoma (61 years old patient)
Hoffmann 1984 (42)	Retrospective cohort study	Adults	59	Complicated appendicitis with appendiceal mass (clinical diagnosis)	5% (3/59)	2 cecal adenocarcinoma, 1 cecal lymphoma

LAMN, Low-grade Appendiceal Mucinous Neoplasm; NET, Neuroendocrine Tumor; SSA, Sessile Serrated Adenoma.

#### 3.2.1 Incidence of neoplasms in uncomplicated acute appendicitis

Primary studies show a low incidence of appendiceal neoplasms in uncomplicated acute appendicitis. In the CODA randomized trial, which enrolled 2,062 adults with CT-confirmed appendicitis without radiologic suspicion of neoplasm, appendiceal tumors were found in 0.6% of patients: 0.7% in the appendectomy group and 0.5% in patients who later underwent appendectomy after initial antibiotic treatment (19).

In the national cohort analysis by Lietzen et al. a tumor prevalence of 0.87% in uncomplicated appendicitis was reported, compared with 3.24% in complicated cases with abscess (30). In another population-based analysis, Salminen et al. documented an incidence of 1.5% among patients with uncomplicated appendicitis, versus 2.4% in complicated appendicitis without abscess and 14.3% in complicated appendicitis with periappendiceal abscess (5). Sugimoto et al. identified appendiceal neoplasms in 22 of 1,277 patients (1.7%), including eight tumors in uncomplicated disease. The overall incidence did not differ significantly after adjustment, although patients aged ≥60 years with appendiceal mass had higher neoplasm rates (23). In a prospective cohort of patients managed non-operatively, Ramadan et al. observed no malignancies during follow-up in individuals with previous uncomplicated appendicitis, whereas patients with prior complicated appendicitis (phlegmon or abscess) had a 5% incidence of malignancy on later evaluation (22).

Additional series that include both uncomplicated and complicated appendicitis report overall neoplasm rates near 1–2%, with lower proportions consistently linked to uncomplicated cases (26, 27). Across these studies, the incidence of appendiceal neoplasms in uncomplicated acute appendicitis remains low, generally within the 0.5–1.5% range.

#### 3.2.2 Incidence of appendiceal neoplasms in appendicitis with abscess

The incidence of appendiceal neoplasms is higher in the setting of complicated acute appendicitis, particularly when an abscess, or inflammatory mass is present. In the registry analysis by Salminen et al., neoplasms were identified in 14.3% of patients affected by complicated appendicitis with periappendiceal abscess (5). This represents the highest incidence reported among primary studies and indicates a distinct risk profile for patients presenting with localized collections. Lietzen et al. also reported a significantly elevated neoplasm rate in complicated appendicitis, with a prevalence of 3.24%. In this study, a separate subgroup analysis of tumor risk associated with periappendiceal abscess on 66 patients showed a significantly higher tumor risk compared with uncomplicated acute appendicitis (4.99% vs. 0.87%). The odds ratio for having an appendiceal tumor in complicated acute appendicitis presenting as periappendiceal abscess was 6.01(CI 95% 4.47–8.08) (30). In the series by Sugimoto et al., 14 of 22 tumors (63.6%) occurred in the complicated appendicitis group (23). Studies focusing on interval appendectomy after treatment of appendiceal abscess or phlegmon report variable neoplasm rates. Carpenter et al. identified tumors in 2.7% of patients undergoing interval appendectomy after complicated appendicitis (38). Wright et al. reported a prevalence of 12.3% in patients who underwent interval appendectomy following initial non-operative management for periappendiceal abscess or phlegmon (34). Similar findings were observed by Son et al., who reported a neoplasm incidence of 12.6% after interval appendectomy in patients with prior complicated disease with phlegmon or abscess (27). In long-term follow-up after non-operative treatment of complicated appendicitis, Ramadan et al. documented a 5% incidence of appendiceal malignancy on delayed evaluation (22).

#### 3.2.3 Preoperative diagnosis of appendiceal neoplasms in acute appendicitis

Appendiceal neoplasms in the setting of acute appendicitis are rarely diagnosed preoperatively and are almost always identified on histopathological examination of the resected appendix. In the CODA trial, 2,062 adults with CT-confirmed acute appendicitis without imaging suspicious for neoplasm were enrolled. Despite this radiologic selection, 12 patients (0.6%) were ultimately found to have appendiceal neoplasms. Using postoperative pathology as the reference, the negative predictive value of CT for appendiceal neoplasm was 99%, whereas the positive predictive value was only 23% (19). Several series explicitly describe tumors as incidental pathological findings. Lietzen et al. reported that tumors were diagnosed at appendectomy for suspected acute appendicitis, with neoplasm present in 1.24% of all appendicitis cases and with higher risk in patients with appendiceal abscess; no routine preoperative neoplasm diagnosis pathway was described (30). Similarly, Son et al. identified appendiceal neoplasms in 1.8% of 2,013 surgically treated adults, with diagnosis based on pathological analysis; preoperative identification was not reported (27). Hayes et al. detected 36 neoplasms (9%) in 402 interval appendectomies for complicated appendicitis (perforation, phlegmon or abscess), again as final pathology findings (24). In the large Finnish periappendiceal abscess cohort, no appendiceal neoplasm was diagnosed preoperatively (5). Naar et al. and Sugimoto et al. evaluated CT features associated with malignancy, including appendiceal diameter and presence of phlegmon, and both showed that certain radiologic findings correlate with increased tumor risk, particularly in older patients; however, neoplasms were still defined by postoperative histology rather than imaging alone (23, 26). In particular, Naar et al. identified the combination of age >40 and an appendiceal diameter >10 mm at the CT scan being associated with a greater than 3-fold increased risk of malignancy in patients presenting with appendicitis.

#### 3.2.4 Histologic types of appendiceal neoplasms

Research studies of patients operated on for acute appendicitis show a broad histologic spectrum, including NET, LAMN and other mucinous lesions, nonmucinous adenocarcinoma, goblet cell tumors, sessile serrated lesions, and adenomas. In uncomplicated appendicitis, adenomas and NET are frequent, with LAMN and adenocarcinoma also present. In complicated appendicitis, particularly with periappendiceal abscess or after interval appendectomy, there is a clear shift toward LAMN and related lesions and adenocarcinoma, while NET and goblet cell tumors remain an important but numerically smaller component.

The most detailed distribution by disease severity derives from the large population-based cohort of Salminen et al. (5). Among 53 patients with periappendiceal abscess and an appendiceal tumor, the predominant histologies were LAMN (n=21) and adenocarcinoma (n=20), followed by adenoma (n=8) and NET (n=5); one patient harbored both LAMN and adenocarcinoma. In 48 uncomplicated appendicitis with tumor, adenoma was most frequent (n=16), followed by NET (n=14), LAMN (n=13), and adenocarcinoma (n=5). In 63 complicated appendicitis without abscess and tumor, adenocarcinoma (n=21) and NET (n=21) were co-dominant, with additional adenomas (n=12) and LAMN (n=9).

These findings are consistent with the single-center series of Sugimoto et al., who identified 22 appendiceal tumors among 1,277 appendectomies. Histology included adenocarcinoma of the appendix (n=6), cecal adenocarcinoma (n=1), metastatic carcinoma (n=2), LAMN (n=5), NET (n=2), neuroendocrine carcinoma (n=1), goblet cell adenocarcinoma (n=2), and adenoma (n=3) (23). In cohorts limited to interval appendectomy after complicated appendicitis, mucinous and adenocarcinomatous histology is prominent. Wright et al. reported 11 neoplasms among 89 interval appendectomies: mucinous neoplasms (n=6), carcinoid/NET (n=4), and adenocarcinoma (n=1) (34). In the Dutch study by de Jonge et al., 11 neoplasms in 7 patients comprised serrated polyp (n=1), LAMN (n=2), grade 1 NET (n=2), goblet cell carcinoids (n=2), adenocarcinomas (n=3), and signet-ring cell carcinoma (n=1) (28). The Peri-APPAC randomized trial by Mällinen et al. identified 11 tumors in patients with periappendiceal abscess managed with interval appendectomy or surgery for recurrence. Reported histologies included multiple LAMN, sessile serrated adenomas, mucinous cystadenoma, goblet cell carcinoid, appendiceal adenocarcinoma, and cecal adenocarcinoma with associated sessile serrated adenoma (8). In the series by Furman et al., 14 neoplasms were identified among 376 appendectomies; 9 (64.3%) were LAMN, and all neoplasms detected after interval appendectomy were mucinous (37).

Hayes et al. reported 36 neoplasms in 402 interval appendectomies (24). LAMN was the most common subtype, whereas all five adenocarcinomas required additional surgery and, in most cases, adjuvant chemotherapy.

#### 3.2.5 The role of age

Multiple cohorts demonstrate that neoplasm prevalence increases markedly with advancing age and is lowest in younger adults. In a population-based analysis, Lietzen et al. reported that patients with appendiceal abscess and neoplasia were older than those with uncomplicated disease and neoplasia (mean 52.9 vs 40.4 years) (30). Similar age distributions were observed in the CODA trial, where most neoplasms occurred in middle-aged or older adults (19). The largest prospective study evaluating age as a risk modifier is the Peri-APPAC-T cohort by Salminen et al. (5), where age was the only independent prognostic factor for neoplasia (OR 1.06 per year; 95% CI 1.04–1.09). Receiver-operating-characteristic analysis identified 35 years as the optimal cutoff for ruling out neoplasia: only one patient younger than 35 years harbored a tumor (a 23-year-old with NET), yielding a sensitivity of 98.1%.

Histologic stratification shows that NET occur in younger adults (median 45 years), whereas LAMN, adenocarcinoma, and adenomas occur in substantially older patients (median 68–76 years). Interval appendectomy cohorts show similar age profiles. Across the studies by Carpenter, Furman, Wright, Mällinen, Al-Kurd, and Mima, the mean age of patients with appendiceal neoplasia ranged from 49 to 62 years (8, 31, 34, 37, 38, 43). Age-specific analyses consistently reported elevated tumor rates in adults >40–50 years, with a further increase in those >70 years. In the series by Son et al., neoplasm incidence was higher in adults >40 years (3.3%) compared with younger patients (0.1%) (27). Pediatric populations show minimal risk. In the cohort described by Hall et al., neoplasms were rare and limited to NET, with no cases of adenocarcinoma (33).

#### 3.2.6 Evidence in children

Children presenting with acute appendicitis, including complicated forms with appendiceal mass or abscess, have a negligible risk of underlying appendiceal neoplasia. Across pediatric cohorts, appendiceal tumors are uncommon and are predominantly low-grade NET, without a consistent association with appendiceal abscess.

Gahukamble et al. evaluated children undergoing delayed appendectomy after appendiceal mass and reported chronic inflammatory or fibrotic changes without neoplastic lesions in all specimens (44). Fouad et al. examined children treated with non-operative management for appendiceal mass, including cases requiring percutaneous drainage, and found no appendiceal neoplasms at interval appendectomy (29).

The CHINA randomized controlled trial compared routine interval appendectomy with observation after successful non-operative treatment of appendiceal mass and reported no missed neoplasms during follow-up in either group (35). Long-term follow-up of this cohort demonstrated no subsequent diagnosis of appendiceal malignancy (36).

Farr et al. reported no tumors identified during follow-up (25). In a contemporary cohort, Hellmann et al. reported that appendiceal tumors in children were rare, occurred as incidental findings after appendectomy, and were limited to NET histology, without association with appendiceal abscess (20). Similarly, Hall et al. reported that pediatric appendiceal neoplasms were almost exclusively NET, typically small and low grade, with no reported cases of adenocarcinoma or mucinous neoplasms in children presenting with appendiceal mass (33). None of the included pediatric studies identified appendiceal abscess as a risk factor for epithelial malignancy.

## 4 Discussion

The present review of the literature examines emerging evidence suggesting that, in a relevant proportion of adult patients with complicated acute appendicitis, an appendiceal abscess may represent the clinical expression of an underlying neoplasm rather than a benign inflammatory process, a concept that remains insufficiently integrated into routine practice. Recognizing this possibility shifts the clinical perspective from viewing appendiceal abscess solely as an infectious condition toward acknowledging its potential role as a marker of occult malignancy in a subset of patients with increased oncologic risk. Although the most frequent neoplasms identified in patients with appendiceal abscess, such as LAMN and low-grade NET, generally carry a favorable prognosis, invasive adenocarcinoma is also reported across several primary studies (45).

However, the presence of high-grade malignancies confirms that the neoplastic spectrum in this setting is not limited to indolent lesions. The complexity of this problem is further heightened by the limited ability of preoperative CT scan to detect appendiceal neoplasms. Across primary studies, tumors were diagnosed almost exclusively on postoperative histopathology, even when high-quality CT imaging revealed no suspicious features. The CODA randomized trial highlights this limitation, reporting a negative predictive value of 99%, but a positive predictive value of only 23% for CT in detecting neoplasia (19). Similar observations were reported in large cohorts of periappendiceal abscess, interval appendectomy and mixed appendicitis, where no neoplasm was identified preoperatively (5, 24, 27, 30).

Because CT cannot reliably exclude malignancy, management must rely on epidemiologic predictors rather than imaging alone. Age consistently emerges as the most powerful predictor of neoplastic risk. Observational studies and interval appendectomy cohorts show that patients with appendiceal tumors are typically middle-aged or older, with mean ages ranging from the late forties to the early sixties (8, 31, 34, 37, 38, 43). The prospective Peri-AppendicitisAcutaTumor (Peri-APPAC-T) cohort study reported that age was the only independent predictor of underlying neoplasia, and a threshold of 35 years identified a subgroup with a markedly increased incidence of malignancy. Only one tumor was found in patients younger than 35 years, and it was a low-grade NET (5). Histologic distributions reinforce this association, as NET predominate in younger individuals whereas LAMN, mucinous lesions and adenocarcinoma are more frequent in older adults, particularly in those presenting with abscess (23, 28, 34).

These findings inform the recommendations of the 2025 WSES Jerusalem Guidelines, which endorse a selective approach to interval appendectomy (46). Adults aged 35 years or older with periappendiceal abscess should undergo structured post-NOM evaluation including CT and colonoscopy, followed by interval appendectomy to ensure oncologic safety. Younger adults do not exhibit the same risk profile and therefore do not require interval appendectomy unless symptoms recur or clinical or radiologic suspicion persists. Children show an even lower incidence of neoplasia, almost exclusively limited to NET (33). Applying adult management strategies to the pediatric population may lead to unnecessary surgery, as appendiceal abscess does not represent a marker of malignancy in children (20, 25, 29, 33, 35, 36, 44). In children surgical intervention should therefore be reserved for recurrence or failure of non-operative management rather than for neoplasm prevention.

For patients with appendiceal abscess, the decision between immediate appendectomy and conservative treatment continues to generate debate. The randomized trial by Mentula et al. showed that laparoscopic appendectomy is safe and feasible when performed by experienced surgeons and reduces readmissions and additional interventions compared with non-operative management (47). However, this must be balanced against the oncologic risk associated with potential rupture of mucinous neoplasms during emergency surgery, with a subsequent risk of peritoneal dissemination. This concern is particularly relevant because mucinous tumors represent a considerable proportion of neoplasms found in abscess patients (5, 23). In settings without advanced laparoscopic expertise, this risk becomes even more pronounced. Since non-operative management has proven highly effective, with success rates exceeding 80% to 90% (48–50), delayed interval appendectomy after six to twelve weeks offers a safer and more controlled environment for definitive surgery, with the eventual possibility of referring the patient to a more skilled surgical team.

Percutaneous drainage adds another layer of complexity. Although drainage improves the success of non-operative management and reduces early recurrence (49, 51), it may theoretically facilitate tumor seeding in the presence of mucinous neoplasia. Drainage should therefore be used selectively in older adults and in patients with atypical imaging findings, with close clinical reassessment once the acute phase resolves.

Interpretation of available data is further complicated by substantial heterogeneity in reported neoplasm incidences. This inconsistency likely reflects variable definitions of complicated appendicitis. Many studies group phlegmon, inflammatory mass, contained perforation and true abscess into a single category, although these entities are biologically and pathologically distinct. The highest tumor incidences are consistently observed in patients with CT-confirmed abscess, as described by Salminen et al. (5), whereas phlegmon is associated with lower risk. Studies that fail to distinguish between these presentations risk diluting the true prevalence of neoplasia in abscess and overestimating risk in other forms of complicated appendicitis.

A further limitation is that nearly all published evidence comes from single-country cohorts, often reflecting local demographics, referral patterns and imaging availability. This limits the external validity of current data and complicates efforts to derive universally applicable risk estimates. An international registry specifically designed to enroll patients with CT-confirmed appendiceal abscess would allow a more accurate assessment of the true global incidence of underlying neoplasia. Moreover, a trans-population registry with standardized radiologic definitions and uniform follow-up protocols would offer an opportunity to validate current age thresholds, to refine management pathways and to mitigate geographic disparities in evidence generation.

Accurate classification of acute appendicitis severity requires preoperative CT imaging. Low-dose CT is now widely considered the diagnostic gold standard and remains essential for distinguishing uncomplicated appendicitis from abscess (52–54). This reliance on CT raises important concerns regarding equity of care because access to advanced imaging varies across health systems. Where CT availability is limited, clinicians may be unable to identify patients with periappendiceal abscess, leading to inappropriate risk stratification and potentially delayed diagnosis of malignancy.

Future research should prioritize standardized imaging-based definitions that clearly separate abscess from phlegmon and should focus on patients with CT-confirmed abscess, who represent the subgroup with the highest oncologic risk. Prospective studies with uniform follow-up protocols, including colonoscopy and interval CT, will be essential to refine risk estimates, guide management strategies and improve equitable access to evidence-based care.

Regarding post-operative strategies for unexpected diagnosis of neoplasia after appendectomy, appendectomy alone is curative for LAMN and low-grade neuroendocrine tumors confined to the appendix with negative margins (45), whereas right hemicolectomy with oncologic lymphadenectomy should be pursued for most cases of appendiceal adenocarcinoma in suitable surgical candidates (55).

In conclusion, appendiceal abscess in adults aged 35 years or older is associated with a clinically relevant risk of underlying neoplasia, most often LAMN and NET, but also invasive adenocarcinoma. Because CT cannot reliably exclude malignancy, interval appendectomy after successful non-operative management remains the safest strategy to achieve diagnostic certainty andxoncologic completeness. An international registry focused on CT-confirmed appendiceal abscess is required to establish accurate incidence estimates and to refine evidence-based management pathways.
